# Good Tolerance of Citrate Accumulation due to Plasma Exchange among Patients with Acute-on-Chronic Liver Failure: A Prospective, Observational Study

**DOI:** 10.1155/2018/4909742

**Published:** 2018-04-18

**Authors:** Yuanji Ma, Yan Xu, Fang Chen, Ying Wang, Lang Bai, Hong Tang

**Affiliations:** ^1^Center of Infectious Diseases, West China Hospital of Sichuan University, Chengdu 610041, China; ^2^Division of Infectious Diseases, State Key Laboratory of Biotherapy, Sichuan University, Chengdu 610041, China

## Abstract

**Aim:**

To assess the tolerance of citrate accumulation due to plasma exchange (PE) among patients with acute-on-chronic liver failure (ACLF).

**Methods:**

A prospective, observational study was conducted among patients with ACLF who received heparin anticoagulation during PE-centered therapy without filtration and dialysis. Citrate accumulation was defined as the value of total calcium (Ca_tot_) to ionized calcium (Ca_ion_) ratio (Ca_tot_/Ca_ion_) greater than or equal to 2.5 (Ca_tot_/Ca_ion_ ≥ 2.5).

**Results:**

Fifty-four patients were enrolled. The mean age and MELD score were 50.0 ± 11.3 years old and 25 ± 7, respectively. Thirty-three patients had liver cirrhosis. The total 3-month survival rate was 57.4% (31/54). The mean Ca_tot_/Ca_ion_ at the time before PE was 2.05 ± 0.14. Ca_tot_/Ca_ion_ ≥ 2.5 occurred in 100.0% (54/54) and 29.6% (16/54) of patients with mean Ca_tot_/Ca_ion_ of 4.34 ± 1.52 and 2.36 ± 0.32 immediately after PE and 1 hour after PE, respectively, and these levels were much higher than those before PE (*p* < 0.01). However, all values returned to lower than 2.5 by the next morning with no difference from those before PE (2.10 ± 0.14 versus 2.05 ± 0.14, *p* > 0.05). Hypocalcemia (ionized calcium) and mild alkalosis were the main metabolic alterations. No symptoms associated with hypocalcemia occurred.

**Conclusions:**

Citrate accumulation is well tolerated by patients with ACLF who receive PE-centered therapy without filtration and dialysis. This study is regeristed with ChiCTR-OOC-17013618.

## 1. Introduction

Acute-on-chronic liver failure (ACLF) remains an important cause of mortality. The accumulation of various toxins and inflammatory cytokines leads to life-threatening complications, including renal failure, altered immune response, hepatic coma, and systemic hemodynamic dysfunction, which eventually culminate in multiorgan failure [1, 2]. Current medical therapy involves the management of the precipitating event and treatment of complications until the liver eventually recovers. Removal of toxins improves the capacity of the liver to regenerate, or artificial liver support system (ALSS) therapy can be a bridge to liver transplantation until a suitable organ is available [2–7].

To adequately maintain extracorporeal circulation, heparin or low-molecular-weight heparin anticoagulation is often used in clinical practice, but its side effects such as bleeding and thrombocytopenia threaten the safety of the patient. In recent years, regional citrate anticoagulation (RCA) has become the preferred anticoagulation method in continuous renal replacement therapy (CRRT) for patients with acute kidney injury (AKI) [8]. Several studies have focused on the safety and efficacy of RCA during blood purification for patients with liver failure and have concluded that RCA seems safe and feasible for these patients [9–16]. However, the blood purification techniques used in these studies, including continuous venovenous hemodialysis (CVVHD) [9, 11, 16, 17], sustained low-efficiency dialysis [12], molecular adsorbent recirculating system (MARS) [10, 14, 15], and fractionated plasma separation and adsorption (FPSA; the Prometheus system) [13], contain a dialysis technique that has the ability to remove citrate. Despite partial removal of the citrate by the dialyzer as a complex bound with ionized calcium (Ca_ion_), a certain amount of citrate enters the systemic circulation, which might lead to citrate accumulation, acid-base imbalance, and hypocalcemia and so on [18].

MARS and FPSA have been widely used for liver failure in Western countries during the past 3 decades, while plasma exchange- (PE-) centered ALSS therapy has been widely used in China for nearly the last 2 decades. With the benefit of having no effect on the blood clotting mechanism in vivo, RCA seems safe and feasible in patients with liver failure who received MARS or FPSA [10, 13–15]. However, without dialysis and filtration techniques, it remains uncertain whether RCA is still effective and safe for patients with liver failure and bleeding tendency or active bleeding who received PE-centered ALSS therapy or not.

Although citrate metabolism is severely impaired and the risk of adverse effects is high in patients with acute liver failure who receive PE therapy with frozen plasma containing citrate [19], it might be not necessarily the same as ACLF for there are big differences between acute liver failure and ACLF [20], and, more importantly, two prospective, controlled studies have found that PE plus standard medical therapy versus standard medical therapy alone could improve the short-term prognosis of patients with acute liver failure [4] and patients with ACLF [3]. Thus, we predict that patients with liver failure still have a certain ability to tolerate and metabolize citrate. However, the tolerance of citrate in patients with ACLF has not been studied in detail. Here, we conducted a prospective, observational study to assess the tolerance of citrate accumulation due to PE among patients with hepatitis B virus (HBV) related ACLF (HBV-ACLF) who received PE-centered ALSS therapy but did not receive filtration or dialysis.

## 2. Materials and Methods

### 2.1. Study Design and Patients

To assess the tolerance of citrate accumulation due to PE among patients with HBV-ACLF, a prospective, observational study was conducted in the Center of Infectious Diseases, West China Hospital of Sichuan University. Patients with HBV-ACLF who received PE-centered therapy for the first session were recruited from March 1, 2017, to June 31, 2017.

**Figure 1:**
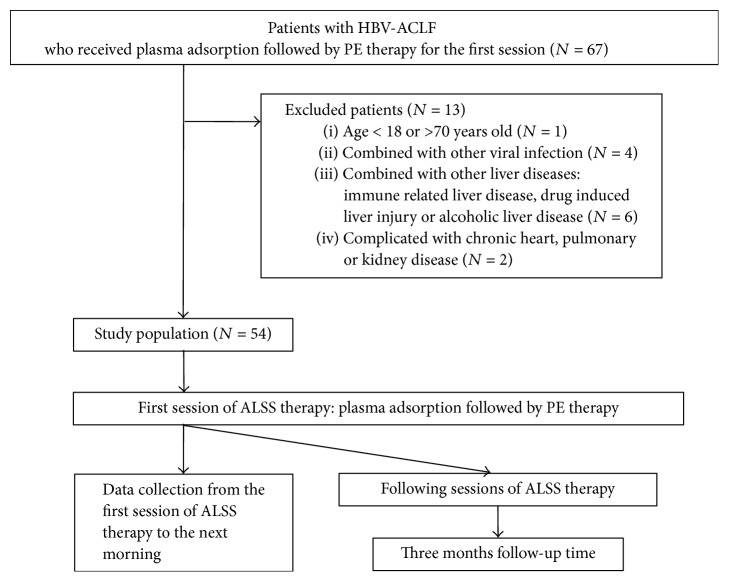
Flow diagram of patient selection and study process. PE, plasma exchange; HBV-ACLF, hepatitis B virus-related acute-on-chronic liver failure; ALSS, artificial liver support system.

Patients with HBV-ACLF who received plasma adsorption followed by PE for the first session of ALSS therapy (a kind of nonbioartificial liver support system widely used in China) were enrolled (Figure 1). The diagnosis of HBV-ACLF was mainly based on the following criteria: (i) preexisting chronic hepatitis B virus infection; (ii) progressive hyperbilirubinemia, defined as a >50% increase in bilirubin or up to a level of >171 *μ*mol/L within 4 weeks; (iii) prothrombin time activity (PTA) ≤ 40% or international normalized ratio (INR) ≥ 1.5. Patients whose age was less than 18 years or more than 70 years were excluded.

Patients were excluded if they had the following diseases: drug-induced liver injury, immune-related liver disease, alcoholic liver disease, and hyperthyroidism, and patients who were pregnant were also excluded. In addition, patients with HBV-ACLF were excluded if they were complicated with other viral infections (including hepatitis A, C, D, or E virus, cytomegalovirus, herpes simplex virus, or human immunodeficiency virus) or had evidence of chronic heart, pulmonary, or kidney diseases. The diagnosis of liver cirrhosis was based on ultrasound and/or computed tomography.

All the patients with HBV-ACLF received standard medication, including antiviral drugs, hepatoprotective agents, and drugs to treat complications. Plasma adsorption followed by PE therapy was performed with heparin anticoagulation. The first session of ALSS therapy was identified as the observational session. Until September 30, 2017, all the patients were followed up for 3 months to identify the status of clinical outcomes.

This study was approved by the Ethics Committee of West China Hospital of Sichuan University. All study components were performed according to the ethical standards laid down in the 1964 Declaration of Helsinki and its later amendments. Written informed consent was obtained from each patient or his/her legal guardian.

### 2.2. ALSS Therapy, Plasma and Citrate

All the study patients received plasma adsorption therapy for 2 hours followed immediately by PE therapy for nearly an hour.

PE-centered ALSS therapy is widely used in China. The dose recommended by the American Society for Apheresis is 1 to 1.5 total plasma volume [21]. In the past, the dose was usually set to one total plasma volume, which was approximately 5% of body weight (approximately 3,000 mL) [22]. Due to the shortage of plasma in recent years and a retrospective study suggesting that plasma adsorption plus PE therapy could reduce the amount of plasma needed for PE therapy without impairing the therapeutic efficiency [23], plasma adsorption plus PE with half the total plasma volume (approximately 1,500 mL) is now widely used for ALSS therapy in China.

Citrate is a regular anticoagulant element in the blood preservation solution. Every 1000 mL of the blood preservation solution consists of sodium citrate 13.2 g, citrate 4.8 g, and glucose 14.7 g. A single blood bag contains 75 mL blood preservation solution. Frozen plasma was provided by the Chengdu Blood Center affiliated to Chengdu Health and Family Planning Commission. Whole blood was collected and stored in a blood bag, and plasma was separated and stored in another blood bag for clinical use. Each patient received 7 to 8 (7.9 ± 0.3) bags of plasma, and the total amount of plasma was 1,500 to 1,550 (1,513 ± 22) mL. Therefore, the mean dose of citrate was approximately 61.3 mmol and the mean concentration of citrate in frozen plasma was approximately 40.5 mmol/L.

**Figure 2:**
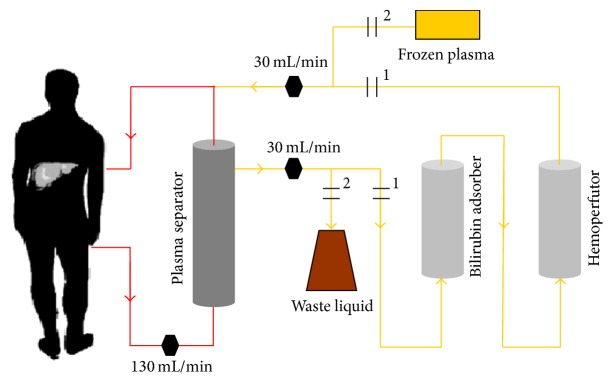
Schematic diagram of the double plasma molecular adsorption system (DPMAS) and plasma exchange (PE). PE ( circuit 2) is initiated immediately when DPMAS (circuit 1) is completed in this study.

Plasma adsorption was combined with a plasma bilirubin adsorption followed by a plasma hemoperfusion (Figure 2), which is referred to as double plasma molecular adsorption system (DPMAS) in China [24]. The extracorporeal blood lines, plasma separator (MICROPLAS MPS05), plasma bilirubin adsorption column (DX350), and plasma hemoperfutor (HA330-II) were manufactured by B.Braun Diapact CRRT, Bellco S.r.l, Biosun Corporation, and Jafron Biomedical Corporation, respectively.

The treatment parameters were set to postdilution fluid replacement for continuous venovenous hemofiltration (CVVH) with blood flow of 130 mL/min, substitution flow of 1800 mL/h, and ultrafiltration flow of 0 mL/h. Therefore, the total time of PE using 1,500 to 1,550 mL plasma for each patient was 50 to 60 min. Patients received an intravenous injection of heparin sodium with an initial dose of 6,250 U at 5 to 10 minutes before plasma adsorption, and those with suspected coagulation would receive an additional heparin sodium injection with a dose of 3,125 U. Patients received 50 mL of 10% calcium gluconate supplement with a speed of 60 mL/h during PE. At the end of ALSS therapy, all patients received an intravenous injection of protamine sulfate with a dose of 50 mg.

### 2.3. Data Collection and Laboratory Examination

The death or survival information of patients was obtained from electronic medical records in the hospital and/or telephone follow-up. If the survival information could not be obtained from these 2 ways, we made the assumption that the patient had died by the follow-up time. The data of clinical characteristics and laboratory results were obtained from electronic medical records in the hospital. The severity of liver failure was rated according to the model for end-stage liver disease (MELD) [25].

Venous blood samples from the observational sessions of ALSS therapy were collected at the time before DPMAS (the time before ALSS therapy), before PE (immediately after DPMAS), immediately after PE (the time after ALSS therapy), 1 hour after PE (1 hour after ALSS therapy), and the next morning. All the samples were sent to the Department of Laboratory Medicine, West China Hospital of Sichuan University, for analysis within 30 minutes. Routine blood parameters, biochemical parameters, and coagulation function were measured by automatic analyzers according to standard laboratory procedures at the time before DPMAS, immediately after PE, and the next morning. Blood gas analysis was performed using the COBAS b 123 system (Roche Diagnostics) at the time before PE, immediately after PE, 1 hour after PE, and the next morning.

### 2.4. Definition

The presence of total calcium (Ca_tot_) to ionized calcium (Ca_ion_) ratio (Ca_tot_/Ca_ion_) greater than or equal to 2.5 (Ca_tot_/Ca_ion_ ≥ 2.5) with or without metabolic acidosis and an enlarged anion gap are the typical manifestations of citrate accumulation. This means that citrate is not being metabolized in a timely manner into bicarbonate, carbon dioxide, and water [9]. Thus, citrate accumulation was defined as Ca_tot_/Ca_ion_ ≥ 2.5 in this study [9, 16, 26].

### 2.5. Statistical Analysis

Quantitative variables were expressed as mean ± standard deviation (SD), and categorical variables as absolute and relative frequencies. One-way ANOVA was performed to calculate differences between quantitative data, while chi-square test or Fisher's exact test was performed to calculate differences between qualitative data. A* p* value of less than 0.05 was considered to indicate statistical significance. All statistical analyses were performed with SPSS Version 17.0 (SPSS Inc.), and the figures were drawn using GraphPad Prism 6 (GraphPad Software Inc.)

## 3. Results

### 3.1. Patients' Characteristics

From March 1, 2017, to June 31, 2017, fifty-four patients with HBV-ACLF who received plasma adsorption followed by PE for the first session of ALSS therapy were enrolled. Of these patients, the mean age was 50.0 ± 11.3 years old, 41 patients were male, 33 patients had a diagnosis of liver cirrhosis, and the mean level of HBV DNA was 3.8 ± 2.2 log IU/mL. The baseline laboratory parameters of patients before the first session of ALSS therapy are summarized in Table 1. The mean levels of total bilirubin, international normalized ratio (INR) of prothrombin time, creatinine, and MELD score were elevated to 408.1 ± 137.1 *μ*mol/L, 2.0 ± 0.6, 101 ± 91 *μ*mol/L, and 25 ± 7, respectively.

### 3.2. Patient Outcomes

During hospitalization, all the patients with HBV-ACLF received standard medication including antiviral drugs, hepatoprotective agents, and drugs to treat complications. Thirty-seven patients underwent right internal jugular vein catheterization, and the remaining patients right femoral vein catheterization. Patients received a total of 203 sessions of ALSS therapy with a mean number of sessions of 3.8 ± 2.0. The immediate effect of the first session of ALSS therapy is summarized in Table 2.

At the final follow-up on September 30, 2017, all enrolled patients had been discharged. The mean length of hospital stay was 26.0 ± 13.4 days. The survival information was available for all. Four patients received living-donor liver transplantation during hospitalization or the 3-month follow-up period, and then we assumed here that they had died. The occurrences of major complications of liver disease (infection, hemorrhage, hepatorenal syndrome, and hepatic encephalopathy) are summarized in Table 3. The total 3-month survival rate was 57.4% (31/54).

### 3.3. Alteration in Calcium Status and Safety of Citrate Accumulation

**Figure 3:**
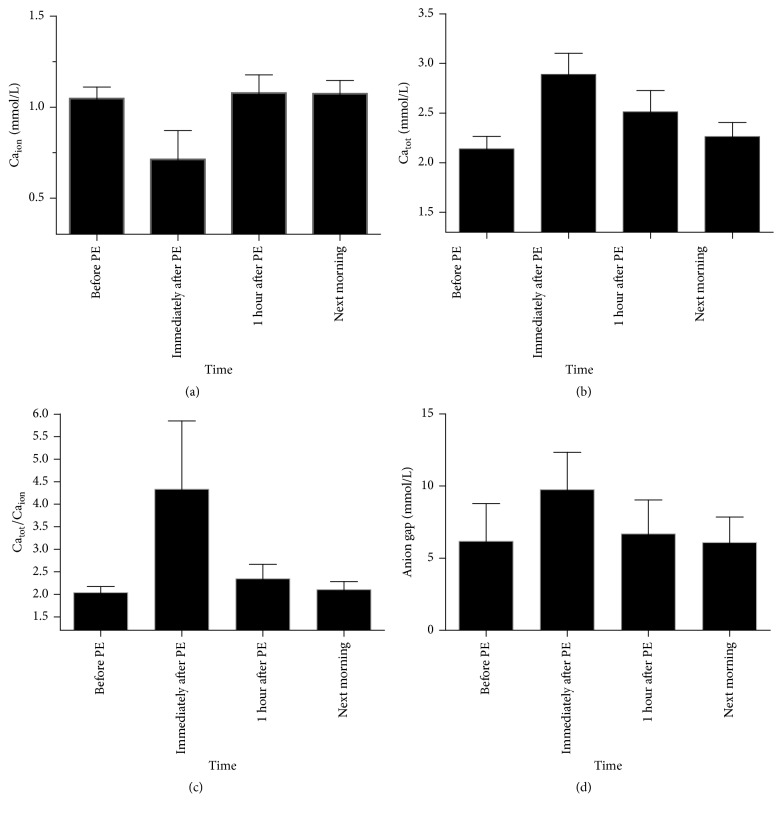
Trends of ionized calcium (Ca_ion_, (a)), total calcium (Ca_tot_, (b)), total-to-ionized calcium ratio (Ca_tot_/Ca_ion_, (c)), and anion gap (d) during and after ALSS therapy for patients with HBV-ACLF.

In this study, we used Ca_tot_/Ca_ion_, an indicator with the cut-off value of 2.5, to assess whether there was citrate accumulation [9, 16, 26], instead of using citrate concentration to indirectly evaluate the presence of citrate accumulation. Changes of Ca_tot_, Ca_ion_, Ca_tot_/Ca_ion_ and anion gap during and after ALSS therapy are summarized in Table 4 and their trends were shown in Figure 3.

The mean level of Ca_ion_ before PE therapy was 1.05 ± 0.06 mmol/L. Although supplied with 50 mL of 10% calcium gluconate during PE, the mean level of Ca_ion_ decreased noticeably to 0.71 ± 0.16 mmol/L immediately after PE, which was much lower than the level before PE (*p* < 0.01). It is worth noting that the mean level of Ca_ion_ returned to normal levels 1 hour after the ALSS therapy and remained stable until the next morning. Although there was an obviously lower level of Ca_ion_ during and after ALSS therapy, none of the patients complained about numbness of the mouth and fingers, nor did we observe any twitching of the calf muscles.

Because of calcium supplements, the mean level of Ca_tot_ immediately after PE was much higher than the level before PE (2.90 ± 0.21 mmol/L versus 2.15 ± 0.13 mmol/L, *p* < 0.01), and the mean level of Ca_tot_ 1 hour after PE was much higher than the level before PE (2.52 ± 0.21 mmol/L versus 2.15 ± 0.13 mmol/L, *p* < 0.01). Although the mean level of Ca_tot_ at the next morning was higher than that before PE (2.27 ± 0.14 mmol/L versus 2.15 ± 0.13 mmol/L, *p* < 0.01), it was noticeably decreased from 2.52 ± 0.21 mmol/L at 1 hour after PE (*p* < 0.01), and the level returned to normal next morning.

Ca_tot_/Ca_ion_ ≥ 2.5, an indicator of citrate accumulation, did not occur before PE, and the mean value of Ca_tot_/Ca_ion_ was 2.05 ± 0.14. Ca_tot_/Ca_ion_ ≥ 2.5 occurred in 100.0% (54/54) and 29.6% (16/54) of patients immediately after PE and 1 hour after PE, respectively, which were much higher than values before PE (*p* < 0.01). The mean values of Ca_tot_/Ca_ion_ immediately after PE and 1 hour after PE were 4.34 ± 1.52 and 2.36 ± 0.32, respectively, which were much higher than that before PE (*p* < 0.01). However, the mean value of Ca_tot_/Ca_ion_ did noticeably decrease 1 hour after PE, and all values returned to lower than 2.5 the next morning. Furthermore, the mean value of Ca_tot_/Ca_ion_ the next morning was similar to that before PE (2.10 ± 0.14 versus 2.05 ± 0.14, *p* > 0.05).

As shown in Table 4 and Figure 3, the mean level of anion gap had similar trends to the mean value of Ca_tot_/Ca_ion_. The mean level of anion gap before PE was 6.2 ± 2.6 mmol/L. The mean level of anion gap was much higher at the time immediately after PE than that before PE (9.8 ± 2.6 mmol/L versus 6.2 ± 2.6 mmol/L, *p* < 0.01). However, the mean level of anion gap returned to the baseline level 1 hour after PE and remained stable the next morning (*p* > 0.05).

### 3.4. Alterations in Acid-Base Status and Lactate

**Figure 4:**
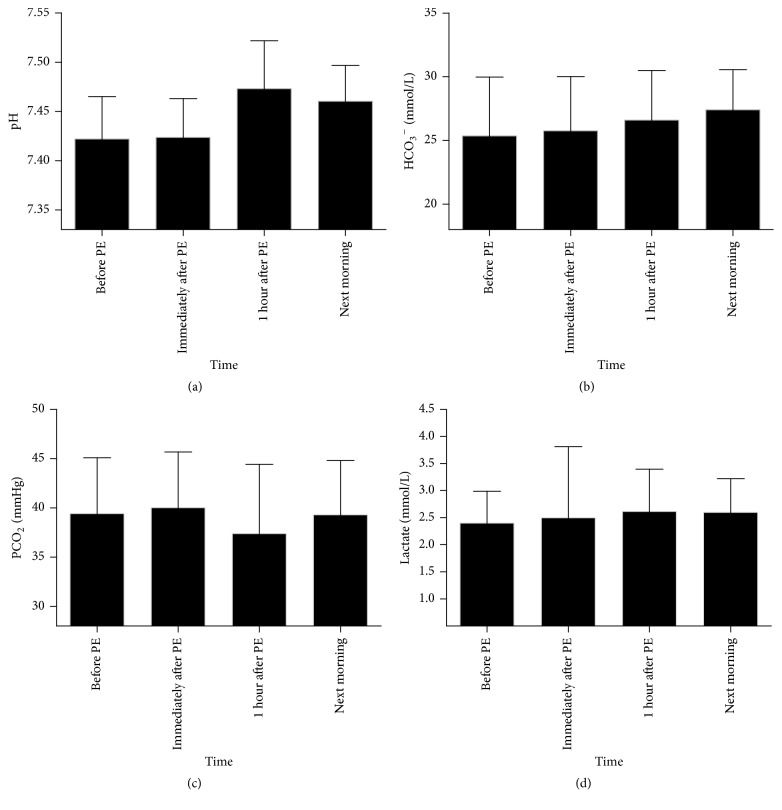
Trends of pH (a), standard bicarbonate (HCO_3_^−^ (b)), partial pressure of carbon dioxide (PCO_2_ (c)), and lactate (d) during and after ALSS therapy for patients with HBV-ACLF.

In the human body, citrate is metabolized into bicarbonate, carbon dioxide, and water. The acid-base status values during and after ALSS therapy are shown in Table 4 and Figure 4.

Although the mean pH immediately after PE was similar to that before PE (7.42 ± 0.04 versus 7.42 ± 0.04, *p* > 0.05), the mean pH noticeably increased 1 hour after PE and the next morning compared with the value before PE (7.47 ± 0.05 versus 7.42 ± 0.04 and 7.46 ± 0.04 versus 7.42 ± 0.04, *p* < 0.01, respectively). However, the mean pH next morning was similar to that 1 hour after PE (7.46 ± 0.04 versus 7.47 ± 0.05, *p* > 0.05).

As shown in Table 4 and Figure 4, the mean level of standard bicarbonate (HCO_3_^−^) increased during and after ALSS therapy. It was higher the next morning than before and immediately after PE and 1 hour after PE (27.4 ± 3.2 mmol/L versus 25.4 ± 4.6 mmol/L, 27.4 ± 3.2 mmol/L versus 25.8 ± 4.3 mmol/L, and 27.4 ± 3.2 versus 26.6 ± 3.9, *p* < 0.05, resp.). The partial pressure of carbon dioxide (PCO_2_) and the lactate level remained stable around the time of ALSS therapy with no difference among the observation time (*p* > 0.05).

### 3.5. Predictors for Citrate Accumulation

Under normal conditions, citrate's half-life is approximately 5 minutes and citrate is metabolized completely within 30 minutes of discontinuing a citrate infusion [27, 28]. However, Ca_tot_/Ca_ion_ ≥ 2.5 occurred in 29.6% (16/54) of our patients 1 hour after PE. Correlation analysis showed that gender (*p* = 0.003), baseline lactate levels (*p* = 0.003), baseline Ca_tot_/Ca_ion_ (*p* = 0.008), baseline Ca_ion_ (*p* = 0.005), and baseline platelet counts (*p* = 0.006) before the ALSS therapy were all predictors for Ca_tot_/Ca_ion_ ≥ 2.5 at 1 hour after PE therapy (Table 5). Multivariate analysis revealed that gender (OR, 0.12; 95% CI, 0.02–0.75; *p* = 0.023), baseline lactate, (OR, 5.53; 95% CI, 1.03–29.57; *p* = 0.046), and baseline Ca_tot_/Ca_ion_ (OR, 5607.59; 95% CI, 2.60–12108390.04; *p* = 0.028) were the independent predictors for citrate accumulation at 1 hour after PE therapy. The highest AUC value regarding citrate accumulation was observed for serum lactate (AUC, 0.750; 95% CI, 0.601–0.899). An increase in Ca_tot_/Ca_ion_ ≥ 2.5 at 1 hour after PE was predicted by the presence of baseline levels of plasma lactate greater than or equal to 2.65 mmol/L (sensitivity 62.5%; specificity 84.2%). The AUC values for gender and baseline Ca_tot_/Ca_ion_ were 0.684 and 0.725, respectively.

## 4. Discussion

In this prospective, observational study, we found that citrate accumulation due to PE occurred in all these patients, and it was still present in 29.6% of these patients at 1 hour after PE. However, all of the levels returned to normal the next morning. Hypocalcemia (ionized calcium) and mild metabolic alkalosis due to PE were the main alterations in calcium and acid-base status. An increase in citrate accumulation at 1 hour after PE was predicted by the presence of baseline levels of plasma lactate greater than or equal to 2.65 mmol/L.

The possible occurrence of citrate accumulation is the main issue for patients with liver failure who are receiving citrate anticoagulation. Several studies have focused on the safety and efficacy of RCA for blood purification in patients with liver failure and AKI and found that RCA seems safe and feasible in these patients [9–17]. Despite partial removal of the citrate by the dialyzer, a certain amount of citrate enters the systemic circulation. Citrate accumulation does occur in 2.3% to 23.2% sessions of CRRT for patients with liver failure and AKI [9, 11, 16, 17] and in 21.4% sessions of MARS therapy for patients with liver failure [10]. In our study, citrate was metabolized by the patients themselves, and citrate accumulation due to PE occurs in all the patients with HBV-ACLF. However, the main acid-base imbalance is mild metabolic alkalosis rather than metabolic acidosis in our study. A similar result was found in a study of patients with cirrhosis, in which the total body clearance of citrate was significantly reduced and the net metabolic changes were quantitatively similar to those in patients with no cirrhosis [29]. Another study of patients with acute liver failure found that citrate metabolism was severely impaired and the risk of adverse effects was high [19]. However, the results from acute liver failure were not necessarily the same as ACLF for there are big differences between them [20]. Our findings suggest that citrate accumulation is well tolerated with good safety among patients with HBV-ACLF, and these patients still have a certain ability to metabolize citrate.

The use of RCA could induce a lower activation of coagulation than both conventional and fractionated heparin, and this lower activation might contribute to an improvement of biocompatibility of extracorporeal circulation [30]. A meta-analysis conducted in adult critically ill patients with AKI who require CRRT reported that RCA is more efficacious in prolonging circuit lifespan and reducing the risk of bleeding with no difference in mortality between the RCA and heparin anticoagulation groups [31]. The benefits mentioned above and good tolerance to citrate seem to somewhat dispel the idea that RCA is contraindicated in patients with liver failure [32]. We think that RCA might be particularly beneficial in patients with impaired coagulation due to liver failure. A similar study reporting dose adaptation and monitoring of Ca_ion_ has also suggested that RCA is feasible for patients with decompensated cirrhosis [29]. However, further research is needed to assess the safety and efficacy of RCA for PE-centered ALSS therapy in patients with HBV-ACLF who do not receive dialysis and filtration.

In this study, we found that gender, baseline lactate levels, and baseline Ca_tot_/Ca_ion_ are independent predictors for citrate accumulation at 1 hour after PE therapy. Similar to our findings, 2 studies have reported that standard liver-function parameters show poor predictive capabilities regarding citrate accumulation in patients with cirrhosis and in patients with liver failure and AKI who received CVVHD [9, 29]. In our study, an increase in citrate accumulation at 1 hour after PE is predicted by the presence of baseline plasma lactate levels greater than or equal to 2.65 mmol/L (sensitivity 62.5%; specificity 84.2%). This finding is similar to that in patients with liver failure and AKI who received CVVHD, in which serum lactate levels greater than or equal to 3.4 mmol/L and prothrombin time activity less than or equal to 26% predict an increase in citrate accumulation with high sensitivity (86% for both lactate and prothrombin time activity) and specificity (86% for lactate; 92% for prothrombin time) [9].

Our study has several limitations. First, we used Ca_tot_/Ca_ion_ instead of direct measurement of plasma citrate concentration to reflect the citrate accumulation. However, citrate metabolism seems not to be restricted to the liver [17], and an upper normal or even toxic level of citrate in the blood is not well established [9]. Being a physiological metabolite, citrate might not be toxic itself but could induce metabolic disorders [9]. Second, we did not directly check the concentration of citrate in frozen plasma. Although we calculated it indirectly by summing up the amount of blood preservation solution the patients used, this might lead to erroneous estimation. Third, we performed blood gas analysis with venous blood samples instead of artery blood samples to reflect acid-base status because of poor clotting function and possible bleeding. However, venous gases are suitable for initial evaluation of acid-base status in critically ill patients [33].

In conclusion, in this prospective, observational study, we found that citrate accumulation due to PE occurred in all the patients with HBV-ACLF who received plasma adsorption and PE but did not receive filtration or dialysis, and it was well tolerated. Further research should be carried out to assess the safety and efficacy of RCA for PE-centered ALSS therapy in patients with HBV-ACLF who do not receive dialysis and filtration.

## Figures and Tables

**Table 1:** Baseline laboratory parameters of patients before the first session of ALSS therapy.

Hemoglobin, g/dL	11.1 ± 2.1
Platelets, ×10^9^/L	126 ± 76
White blood cells, ×10^9^/L	8.2 ± 6.5
INR	2.0 ± 0.6
Total bilirubin, *μ*mol/L	408.1 ± 137.1
Direct bilirubin, *μ*mol/L	307.2 ± 107.9
Alanine transaminase, IU/L	200 ± 232
Aspartate transaminase, IU/L	177 ± 178
Alkaline phosphatase, IU/L	223 ± 320
gamma-Glutamyl transferase, IU/L	141 ± 276
Albumin, g/L	32.1 ± 4.4
Globulin, g/L	28.2 ± 8.2
Total bile acid, *μ*mol/L	290.7 ± 78.6
Creatinine, *μ*mol/L	101 ± 91
Ammonia, mmol/L	76 ± 35
Sodium, mmol/L	133.9 ± 5.3
Potassium, mmol/L	3.4 ± 0.6
Chloride, mmol/L	97.1 ± 6.4
MELD scores	25 ± 7

ALSS, artificial liver support system; MELD, model for end-stage liver disease; INR, international normalized ratio; measurement data are represented as mean ± SD.

**Table 2:** Immediate effect of the first session of ALSS therapy.

	Rate of reduction between pre- and post-ALSS therapy (%)	Rate of reduction between pre-ALSS therapy and the next morning (%)
Hemoglobin	12.1 ± 6.3	3.3 ± 5.9
Platelets	20.0 ± 18.1	17.4 ± 15.3
White blood cells	−7.3 ± 31.6	7.1 ± 21.5
INR	12.7 ± 17.8	9.4 ± 15.2
Total bilirubin	49.1 ± 6.7	14.1 ± 14.8
Direct bilirubin	47.5 ± 7.2	16.8 ± 9.8
Alanine transaminase	36.9 ± 12.2	27.4 ± 13.5
Aspartate transaminase	30.4 ± 57.8	17.1 ± 11.3
Alkaline phosphatase	24.9 ± 33.6	3.2 ± 61.3
gamma-Glutamyl transferase	31.2 ± 14.1	20.0 ± 18.8
Albumin	5.6 ± 7.6	−2.2 ± 8.9
Globulin	13.4 ± 12.3	12.7 ± 8.6
Total bile acid	19.9 ± 14.1	28.5 ± 14.4
Creatinine	7.7 ± 15.4	3.2 ± 16.6
Ammonia	16.3 ± 36.3	−32.5 ± 68.3
Sodium	−3.1 ± 2.5	−1.9 ± 2.4
Potassium	−6.3 ± 10.0	−10.6 ± 14.0
Chloride	−1.2 ± 4.7	−0.3 ± 4.5
MELD score	21.4 ± 13.4	9.2 ± 10.2

ALSS, artificial liver support system; MELD, model for end-stage liver disease; INR, international normalized ratio; measurement data are represented as mean ± SD.

**Table 3:** Disease state during hospitalization and short-term prognosis.

Hospital stay, days	26.0 ± 13.4
Spontaneous bacterial peritonitis, yes/no	30/24
Infection, yes/no	16/38
Hemorrhage, yes/no	7/47
Hepatorenal syndrome, yes/no	12/42
Hepatic encephalopathy, yes/no	15/39
Three-month survival, yes/no^★^	31/23

ALSS, artificial liver support system. ^★^Four patients received living donor liver transplantation during hospitalization or the 3-month follow-up period, we assume here that they have died. Measurement data are represented as mean ± SD. Enumeration data are represented as frequencies.

**Table 4:** Calcium and acid-base status during and after ALSS therapy.

Time	Before PE	Immediately after PE	1 hour after PE	Next morning^★^
Ca_tot_, mmol/L	2.15 ± 0.13^§^	2.90 ± 0.21^#§^	2.52 ± 0.21^#§^	2.27 ± 0.14^#^
Ca_ion_, mmol/L	1.05 ± 0.06	0.71 ± 0.16^#§^	1.08 ± 0.10	1.08 ± 0.06
Ca_tot_/Ca_ion_	2.05 ± 0.14	4.34 ± 1.52^#§^	2.36 ± 0.32^#§^	2.10 ± 0.14
Ca_tot_/Ca_ion_ ≥ 2.5, yes/no	0/54	54/0^#§^	16/38^#§^	0/54
Anion gap, mmol/L	6.2 ± 2.6	9.8 ± 2.6^#§^	6.7 ± 2.3	6.1 ± 1.8
pH	7.42 ± 0.04^§^	7.42 ± 0.04^§^	7.47 ± 0.05^#^	7.46 ± 0.04^#^
HCO_3_^−^, mmol/L	25.4 ± 4.6^△^	25.8 ± 4.3	26.6 ± 3.9	27.4 ± 3.2^*※*^
PCO_2_, mmHg	39.5 ± 5.7	40.1 ± 5.6	37.5 ± 7.0	39.4 ± 5.5
Lactate, mmol/L	2.4 ± 0.6	2.5 ± 1.3	2.6 ± 0.8	2.6 ± 0.6

ALSS, artificial liver support system; PE, plasma exchange; Ca_tot_, total calcium; Ca_ion_, ionized calcium; Ca_tot_/Ca_ion_, total-to-ionized calcium ratio; HCO_3_^−^, standard bicarbonate; PCO_2_, partial pressure of carbon dioxide. ^★^The interval time between the time after PE therapy and the next morning is 16.5 ± 1.9 hours. Compared with the data at the time before PE, ^*※*^*p* < 0.05, ^#^*p* < 0.01, the others, *p* > 0.05. Compared with the data at the next morning, ^△^*p* < 0.05, ^§^*p* < 0.01, the others, *p* > 0.05. Measurement data are represented as mean ± SD. Enumeration data are represented as frequencies.

**Table 5:** Univariate analysis and multivariate analysis of predictors for citrate accumulation at 1 hour after ALSS therapy.

	Correlation coefficient	*p*	Regression coefficient	Standard error	*p*	Odds ratio	95% CI
Gender	0.393	0.003	−2.130	0.938	0.023	0.12	0.02–0.75
Baseline lactate	0.396	0.003	1.710	0.856	0.046	5.53	1.03–29.57
Baseline Ca_tot_/Ca_ion_	0.356	0.008	8.632	3.917	0.028	5607.59	2.60–12108390.04
Baseline Ca_ion_	−0.380	0.005	1.974	8.144	0.809	7.20	0.00–61574425.76
Baseline platelets	−0.368	0.006	0.000	0.006	0.873	1.00	0.99–1.01

ALSS, artificial liver support system; Ca_tot_, total calcium; Ca_ion_, ionized calcium*; *Ca_tot_/Ca_ion_, total-to-ionized calcium ratio.
